# Complement Therapeutics in Autoimmune Disease

**DOI:** 10.3389/fimmu.2019.00672

**Published:** 2019-04-03

**Authors:** Joshua M. Thurman, Roshini Yapa

**Affiliations:** Department of Medicine, University of Colorado School of Medicine, Aurora, CO, United States

**Keywords:** complement, autoimmunity, antibody, immune complex, therapeutic

## Abstract

Many autoimmune diseases are characterized by generation of autoantibodies that bind to host proteins or deposit within tissues as a component of immune complexes. The autoantibodies can activate the complement system, which can mediate tissue damage and trigger systemic inflammation. Complement inhibitory drugs may, therefore, be beneficial across a large number of different autoimmune diseases. Many new anti-complement drugs that target specific activation mechanisms or downstream activation fragments are in development. Based on the shared pathophysiology of autoimmune diseases, some of these complement inhibitory drugs may provide benefit across multiple different diseases. In some antibody-mediated autoimmune diseases, however, unique features of the autoantibodies, the target antigens, or the affected tissues may make it advantageous to block individual components or pathways of the complement system. This paper reviews the evidence that complement is involved in various autoimmune diseases, as well as the studies that have examined whether or not complement inhibitors are effective for treating these diseases.

## Introduction

Autoimmune diseases are conditions in which immunologic tolerance for specific self-proteins is lost, and the adaptive immune system orchestrates injury of organs expressing those proteins. The skin, joints, and kidneys are commonly affected, but any organ in the body can be the target of autoimmunity. Most autoimmune disorders are associated with autoantibodies that are reactive against self-proteins. Systemic lupus erythematosus (SLE), for example, is associated with autoantibodies to nuclear antigens as well as many other intracellular and extracellular antigens (1, 2). Myasthenia gravis is associated with autoantibodies specific for the acetylcholine receptor (AChR). Although not all of the identified autoantibodies have been established as causative of disease, in some cases experiments have convincingly shown that the autoantibodies replicate the disease when transferred into animals that express the target antigen. In membranous nephropathy, for example, there are reported cases in which passage of autoantibodies from mother to fetus have caused antenatal disease (3).

Autoimmunity can be regarded as having two components: (1) the loss of tolerance to self-antigens, and (2) downstream immune-mediated effector mechanisms of injury. Autoantibodies may contribute to disease pathogenesis via several effector mechanisms. They can crosslink target antigens or activate receptors directly on cell surfaces. In Grave's disease, for example, autoantibodies to the thyrotropin receptor trigger release of thyroid hormone, and in myasthenia gravis antibodies to the AChR can cross-link the receptor (4, 5). The inflammatory effects of autoantibodies can also be mediated through ligation of Fc receptors on leukocytes. In some models of autoimmunity this accounts for nearly all of the downstream tissue injury (6). Autoantibodies can also directly damage target tissues, and complement activation is a downstream mediator of this injury in some diseases.

Antibodies bound to cell surface antigens activate complement on the target cells, usually through activation of the classical pathway. Immune-complexes (ICs) can also deposit in small capillaries where they activate complement on bystander cells. Once activated, the complement cascade generates multiple different biologically active fragments. C3a and C5a contribute to chemoattraction and activation of leukocytes (7). C3 fragments (C3b, iC3b, C3dg) fixed to host tissues can activate leukocytes through ligation of complement receptors (CRs) 1–4 (8, 9). C5b-9 causes direct cell activation and cytotoxicity. Although most C5b-9 forms directly on cell membranes, it can also insert into nearby cells causing “bystander” injury (10). Thus, once the complement cascade is activated within a tissue it can trigger multiple local and systemic effects.

Given the prevalence of autoantibodies in the various autoimmune and rheumatic diseases, complement inhibition holds promise as an effective strategy for blocking multiple pathways of injury common to these diseases. In many of the diseases there is also pre-clinical or biomarker evidence to support the use of anti-complement therapeutics. Therapeutic complement inhibitors may be effective at blocking direct tissue injury by the complement cascade. Consequently, they may have a role in rapidly reducing tissue inflammation while other immunosuppressive drugs to block the adaptive immune response. Complement inhibitors may also reduce the adaptive immune response by decreasing stimulation of dendritic cells, T cells, and B cells via the complement receptors (11). This class of drugs could therefore be effective in patients with acute disease flares, but also for chronic treatment of these diseases.

A large number of anti-complement drugs are in development, providing tools for blocking all complement activity, specific activation pathways, or isolated complement fragments (12, 13). Intuitively, drugs that block the classical pathway should be beneficial in antibody-mediated diseases. The classical pathway also helps to prevent autoimmunity and to solubilize ICs, however, so drugs that block downstream targets within the complement cascade while leaving the early classical pathway intact may be preferable in some diseases (discussed below). Drugs that block specific complement fragments, such as C5a receptor (C5aR) antagonists, may also have fewer side effects than drugs that more completely block the complement system. It is also noteworthy that complement activation is not always an important component of disease pathogenesis, even in models in which ICs and complement are deposited within tissues. In some models this is due to the effects of other pathways, such as signaling through Fc receptors (6). In other instances this may be due to intrinsic differences in susceptibility or resistance of target tissues to injury (14). Given these considerations, a benefit to complement inhibition cannot simply be inferred from detection of autoantibodies or complement activation. The efficacy of complement inhibition in autoimmune diseases needs to be studied on a case-by-case basis.

## Systemic Autoimmune Diseases

### Systemic Lupus Erythematosus

SLE is the paradigmatic autoimmune disease. It is a complex disorder characterized by formation of autoantibodies to multiple different nuclear antigens, including DNA, histones, and ribonucleoproteins. There is also evidence that autoantibodies react with antigens expressed in specific tissues, including alpha-enolase, annexin A1, and annexin A2 (15, 16). SLE frequently affects the joints, skin, and kidneys, but essentially any organ can be affected. Organ involvement is usually associated with the deposition of ICs and complement activation. Other downstream mediators of immunologic injury have been identified, however, including cytokines and chemokines, Fc receptor ligation, cellular infiltrates, and toll-like receptor activation (6, 17).

#### The Role of Complement in SLE

**Figure 1:**
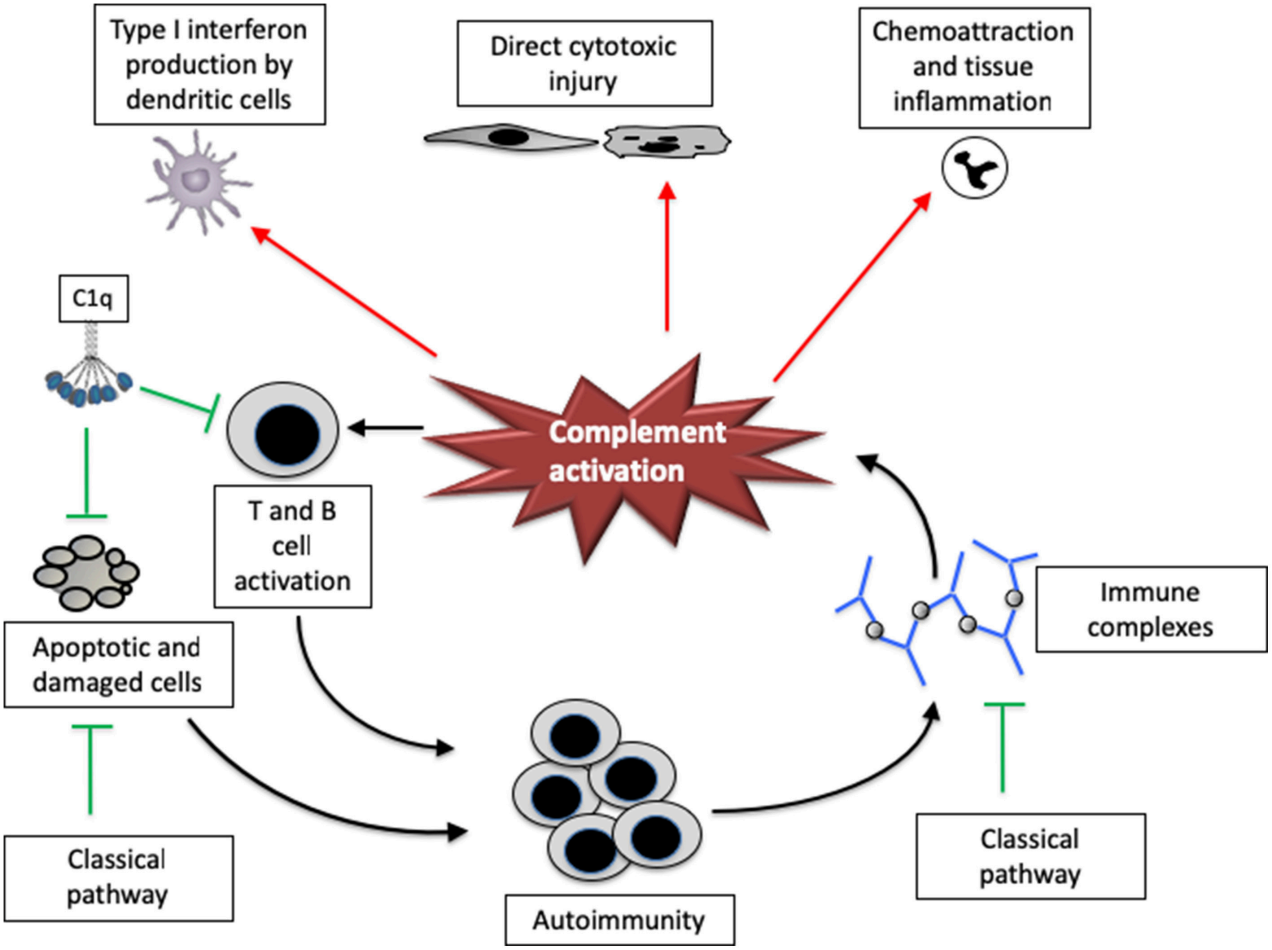
The complement system has both pathogenic and protective roles in systemic lupus erythematosus. Complement proteins have multiple functions that affect lupus, both positively and negatively. The classical pathway may help facilitate the removal of apoptotic and damaged cells as well as immune complexes, reducing the risk of developing autoimmunity to nuclear components. C1q may also directly reduce CD8 T cell activation, thereby attenuating downstream immunity and autoantibody generation. Once activated, however, the complement system can promote tissue injury and disease severity. The complement system can directly cause cytotoxic injury in tissues, and also increases inflammation by attracting leukocytes and inducing interferon production by dendritic cells. Complement fragments may also increase T cell and B cell responsiveness, increasing the response to autoantigens.

The complement system plays a paradoxical role in the pathogenesis of SLE—it seems to lower the risk of developing SLE while also mediating end organ injury in the disease (Figure 1). Congenital deficiency of classical pathway proteins are strong risk factors for developing SLE, presumably because they help protect against autoimmunity. Individuals with deficiencies of C1q, C1s, C1r, C2, and C4, for example, are all at increased risk of the disease (18).

**Figure 2:**
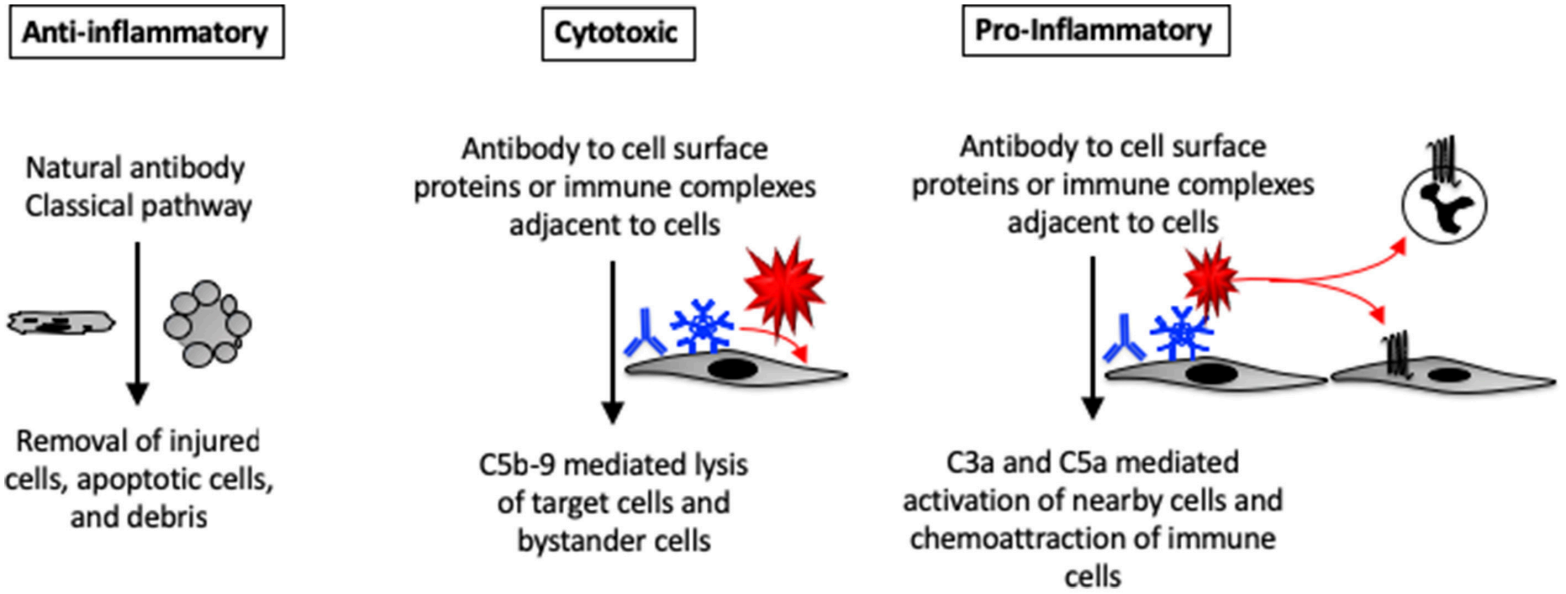
Pro- and anti-inflammatory effects of complement activation in tissues. Complement proteins can directly bind to injured and apoptotic cells. The complement system can also be activated by natural antibodies bound to injured cells. Autoantibodies that are bound to self-antigens can activate complement directly on the cell surface or on immune complexes deposited near the cells. C5b-9 formed on the cell surface can lead to cell activation or lysis. C3a and C5a generated within tissues can promote tissue inflammation by activating nearby cells (which may then secrete cytokines and chemokines) or by causing infiltration of the tissue by circulating leukocytes.

Classical pathway proteins may be protective against SLE though several mechanisms. C1q and C4 opsonize apoptotic cell debris and ICs, and facilitate their clearance [Figure 2, and (19)]. C1q binds directly to molecules displayed on the surface of apoptotic bodies, including DNA and phosphatidylserine (20, 21). Mice with targeted deletion of the *C1qa* gene have a greater abundance of apoptotic bodies in the kidney than control mice, and they develop auto-antibodies to nuclear antigens (22). C1q deficient mice on a pure C57BL/6 background do not develop disease, however, demonstrating that other background factors are also important (23). A recent study also demonstrated that C1q can directly modulate CD8 T cell metabolism via receptors on the cell surface, reducing the response of the cells to self-antigens (24). In this study, C1q deficiency increased renal pathology in a CD8-dependent fashion. Work in animals demonstrates that C4 helps to maintain immune tolerance to self-antigens (25). An intact classical pathway, therefore, may reduce the abundance of nuclear proteins in the extracellular space and also suppress the immune response to these proteins.

Although the classical pathway helps to prevent development of SLE, the complement system is also an important mediator of tissue injury. Complement proteins are seen in kidney biopsies from patients with lupus nephritis and co-localize with deposited ICs (26). Glomerular complement deposits are associated with a worse prognosis (27), suggesting that complement activation contributes to tissue injury. Work in mouse models of lupus-like disease demonstrate that deficiency of classical pathway proteins is not protective [consistent with a protective role for these proteins (28)], whereas deficiency of alternative pathway proteins is protective (29, 30). Several therapies have been shown effective in preclinical studies using models of lupus-like disease, including agents that block the alternative pathway (31, 32), activation at the level of C3 (33), C3a activity (34), and C5a activity (35). Furthermore, treatment with an inhibitory antibody to mouse C5 for 6 months improved renal function and survival in a mouse model of disease (36).

Based on the complex role of complement in SLE, a clinical trial of a complement inhibitory drugs in SLE patients must be designed with equipoise. The treatment should probably not block the classical pathway upstream of C4, even though this is an IC-mediated disease characterized by classical pathway activation.

#### Therapeutic Complement Inhibitors in SLE

A clinical trial of eculizumab in patients with lupus nephritis was planned, but was canceled after enrolling only one patient (37). Eculizumab has been used off-label in patients with proliferative lupus nephritis, and it may be effective in patients with severe disease (38, 39). Several papers have also reported use of eculizumab in SLE patients with thrombotic microangiopathy (40, 41). Apellis is a company that is developing an agent (APL-2) that blocks complement activation at the level of C3. The company is currently conducting a phase 2 trial of the drug that will include patients with lupus nephritis.

### Anti-neutrophil Cytoplasmic Antibody Vasculitis

Anti-neutrophil cytoplasmic antibody (ANCA) vasculitis comprises a group of severe diseases that can affect many organs, including the lungs and upper airways, kidneys, skin, and joints. ANCA associated vasculitis (AAV) is often referred to as “pauci-immune” because immunoglobulin and complement proteins are not prominently seen in tissue biopsies. These diseases are antibody-mediated, however, and the ANCA can cause disease when injected into rodents (42). Antibodies and complement fragments probably function on the surface of neutrophils, and thus are not abundantly deposited within affected tissues. That said, complement fragments are frequently present in tissues biopsies, even if they are less abundant than in IC deposition diseases such as SLE (43).

#### The Role of Complement in ANCA Vasculitis

The important role of the complement system in AAV was revealed by an elegant series of experiments using a murine model of the disease. Using mice with various complement deficiencies, investigators found that factor B deficiency was protective but that C4 deficiency was not (44). Therefore, even though this an antibody-mediated disease, the alternative pathway appears to be more important than the classical pathway for disease pathogenesis. C5 deficiency is also protective in this model but C6 deficiency was not, suggesting that C5a is more important than C5b-9 formation in disease pathogenesis. Although referred to as “pauci-immune,” there is clinical evidence of complement activation in human patients with AAV. Glomerular C3 deposits can be seen in more than 40% of patients (45). Complement activation fragments are also elevated in plasma and urine of patients with AAV, including the fragment Bb [an alternative pathway activation fragment (46)].

#### Therapeutic Complement Inhibitors in ANCA Vasculitis

In the murine model of AAV, a monoclonal antibody to C5 (BB5.1) and an orally delivered small molecule C5a receptor antagonist (CCX168) were both nearly completely protective (47, 48). These results demonstrated that that C5a blockade is sufficient for blocking the complement effects in AAV. A subsequent phase 2 study of CCX168 (conducted by the company ChemoCentryx) tested whether the drug could be used in lieu of corticosteroids (49). This study included a group of patients that received cyclophosphamide and CCX168 without corticosteroids. That treatment approach was non-inferior when compared to standard therapy (corticosteroids + cyclophosphamide). A phase 3 study is currently underway evaluating the efficacy of the drug in combination with rituximab or cyclophosphamide. The study will compare CCX168 to prednisone in combination with the same other immunosuppressive agents.

### Antiphospholipid Antibody Syndrome

Anti-phospholipid antibodies (APLA) are autoantibodies that bind to endothelial cells, triggering thrombosis. The antiphospholipid syndrome (APS) is diagnosed by detection of these antibodies in patients who have had a thromboembolic event (50). The antibodies associated with APS can be reactive to cardiolipin, β2-glycoprotein 1, or they can be detected as “lupus anticoagulants.” The lupus anticoagulant assay detects the antibodies indirectly, through the interference of clotting on phospholipid surfaces *in vitro*.

#### The Role of Complement in APS

Clinically, APS is associated with thrombosis or fetal loss. In a passive transfer model in rats, anti-β2 glycoprotein I antibodies caused thrombosis in rats only if they had previously been treated with lipopolysaccharide (51). Complement deficiency or inhibition prevented thrombosis in this model. A murine model of fetal loss caused by passive transfer of antiphospholipid antibodies also demonstrated a role for complement activation in the disease (52). The disease requires an intact alternative pathway, and treatment of mice with an inhibitory antibody to factor B protected them from fetal loss (53). Use of a C5a receptor antagonist was also protective in this model (54). There is evidence of complement consumption in patients with APS. Plasma C3 and C4 levels are lower than in healthy and disease-control subjects, and complement activation fragments are elevated (55). Detailed examination of a patient with APS has also demonstrated elevated levels of circulating complement fragments and also complement fragments deposited within an area of arterial thrombosis (56).

#### Therapeutic Complement Inhibitors in APS

The standard therapy for APS is anticoagulation. Catastrophic APS (CAPS) is a syndrome in which patients have thrombosis of multiple organs, and eculizumab has been used in patients with disease refractory to anticoagulation or conventional immunosuppression (57). There are also published case series of CAPS patients who were treated with eculizumab to prevent disease recurrence in renal transplants (58). An open-label phase 3 trial of eculizumab for preventing recurrence of CAPS in renal transplant patients was started, although it is apparently not recruiting patients currently.

## Organ Specific Autoimmune Diseases

### Membranous Nephropathy

Membranous nephropathy (MN) is a form of kidney disease in which ICs deposit in the glomerular capillary wall between the podocyte and the basement membrane (“subepithelial ICs”). The podocyte is a specialized cell that sits on the urine side of the glomerular capillary, and it provides a barrier to leakage of cells and protein into the urine. The subepithelial ICs cause podocyte injury, thereby disrupting the filtration barrier. This leads to the leakage of protein into the urine, and over time it can cause irreversible glomerular damage and a loss of kidney function.

In most cases of MN, autoantibodies bind to proteins expressed on the podocyte surface (referred to as primary MN). In some settings, however, antigens from elsewhere in the body become trapped under the podocyte. Antibodies can then bind the planted antigens *in situ* (referred to as secondary MN). Primary MN is an autoimmune disease in which the antibodies bind to self-proteins expressed by the podocyte, and several different target podocyte antigens have been identified. The first podocyte antigen identified in MN was neutral endopeptidase (NEP) (3). Mothers who are genetically deficient in NEP can be exposed to the protein during pregnancy, eliciting an immune response. During subsequent pregnancies, the maternal anti-NEP antibodies are transferred to the infant, leading to transient MN in the baby.

The most common target antigen in primary MN is the M-type phospholipase A2 receptor (PLA2R), a transmembrane protein expressed by the podocyte (59). Antibodies to PLA2R are seen in 70–80% of cases of MN. Approximately 5% of patients with primary MN have autoantibodies against thrombospondin type-1 domain-containing 7A (THSD7A) (60). It is not known why immune tolerance to these podocyte proteins is lost in patients with MN, but the disease is associated with certain HLA alleles (61), and the autoantibodies bind to specific epitopes on the target proteins (62, 63).

Autoantibodies to several intracellular proteins have also been found, including antibodies reactive with alpha enolase, aldose reductase, and manganese superoxide dismutase (SOD2) (64, 65). Initial injury to the podocyte may be required to cause release of these proteins into the extracellular space. Secondary MN is associated with numerous different systemic diseases, including SLE, several types of cancer, and various infections. Cancer-associated antigens and infection-associated antigens have been eluted from the glomerular deposits in cases of secondary MN, suggesting that these proteins reach the kidney via the circulation and become trapped under the podocytes. Antibodies then bind to the target antigens, and presumably injure the podocyte through the same inflammatory mechanisms as in primary disease.

#### The Role of Complement in MN

**Figure 3:**
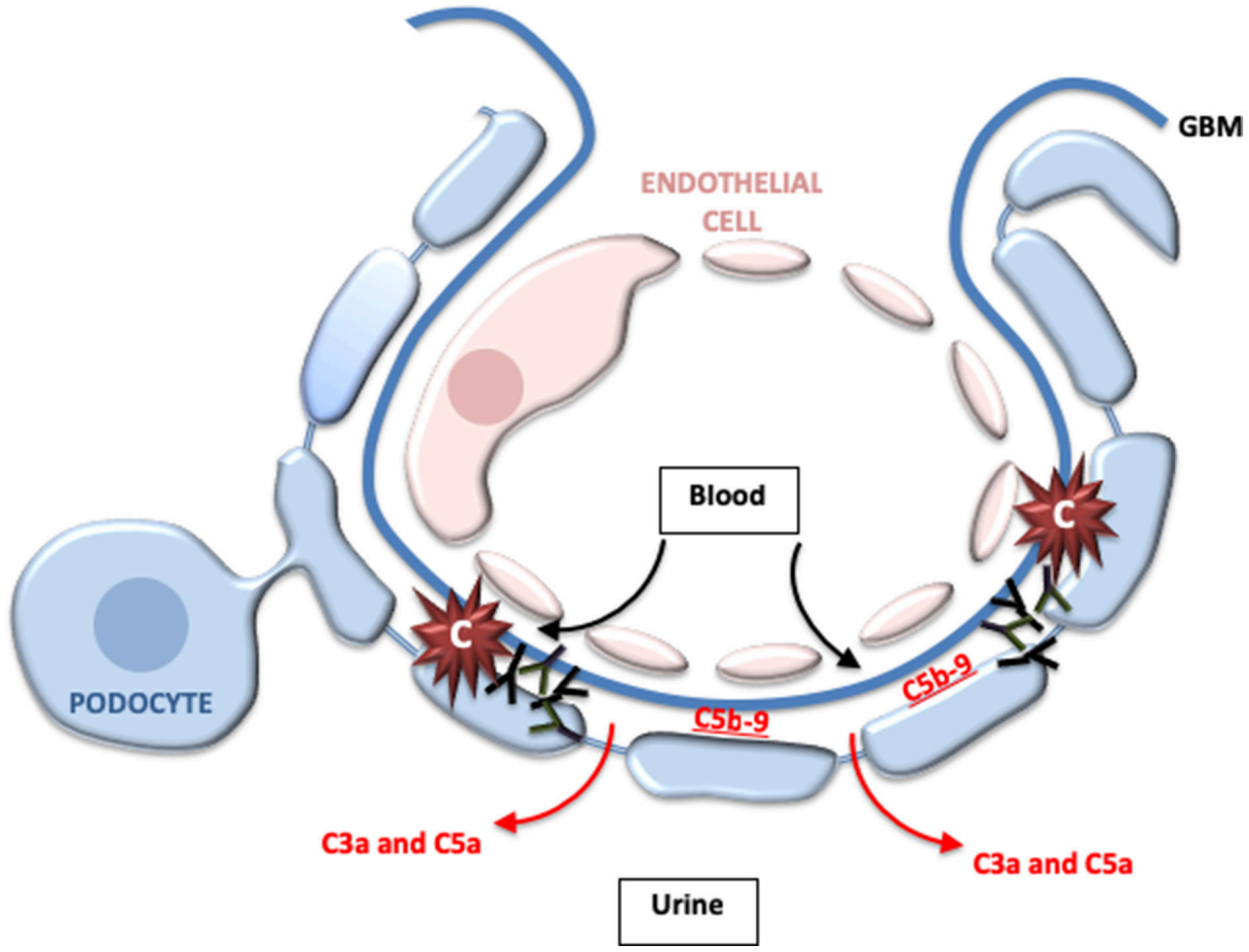
Complement activation may directly injure the podocytes in membranous nephropathy. In membranous nephropathy, immune complexes are located between the glomerular basement membrane (GBM) and the podocyte foot processes. Activation at this location generates C5b-9, which may directly damage podocytes. Because of the location of the immune complexes, however, most of the C3a and C5a that is generated probably passes into the urine and does not enter the bloodstream. This may explain why leukocytes do not typically infiltrate the glomeruli in this disease.

The subepithelial ICs in MN activate complement either directly on the surface of podocytes or immediately adjacent to the cells, and C3 is detected in most biopsies. The C3a and C5a generated by complement activation at this location likely pass directly into the urine, explaining why neutrophils are not usually seen in kidney biopsies (Figure 3). Similarly, histologic hallmarks of inflammation are not usually seen in biopsies. C5b-9 that is formed on the podocyte, on the other hand, can directly injure the cell. Early studies using animal models of MN indicated that it is a complement dependent disease (66). Furthermore, animals deficient in C6 are protected from injury, further implicating C5b-9 in podocyte injury and the pathogenesis of the disease (67, 68).

Interestingly, in primary MN the autoantibodies are predominantly of the IgG4 subclass, which does not activate the classical pathway of complement (69). Furthermore, C1q is not detected in most biopsies, whereas C4 is usually detected. These observations suggest that the autoantibodies may activate complement through the mannose binding lectin (MBL) pathway. Investigators have proposed that the carbohydrate side chains of IgG4 may bind to MBL and activate the lectin pathway (70). There are, however, reports of patients with congenital MBL deficiency who have developed MN (71). These patients had C3 deposits in the absence of C1q or C4 deposits, indicating that activation can occur solely through the alternative pathway and that the MBL pathway is not always required. Further complicating things, some IgG1 and IgG3 autoantibodies are detected in the circulation and in the glomerular deposits in primary MN (59, 72). IgG1, IgG2, and IgG3 are also usually seen in biopsies from patients with secondary MN (73, 74). Therefore, although complement is activated in both primary and secondary MN, the mechanisms may vary from patient to patient.

#### Therapeutic Complement Inhibitors in MN

Based on the pre-clinical evidence that C5b-9 formation is important to the pathogenesis of MN, a clinical trial was conducted in which 122 MN patients were treated with eculizumab or placebo for 16 weeks (75). Treatment with eculizumab was not associated with a significant reduction in proteinuria, indicating that complement inhibition may not be of benefit in the disease. It is possible, though, that the lack of benefit was due to the short duration of treatment. It is also noteworthy that the doses of eculizumab used in the study may not have completely blocked terminal complement activation (76). Many new complement inhibitors are currently in development (13, 77), and several ongoing clinical trials are including patients with MN. Omeros has developed an inhibitory antibody to MASP2 (OMS721). OMS721 and APL-2 (a C3 activation inhibitor) are currently being tested in Phase 2 studies that include patients with MN (13).

### Myasthenia Gravis

Myasthenia gravis (MG) is an autoimmune disease caused by antibodies to proteins expressed at the neuromuscular junction, most commonly the AChR (78). The antibodies can block the function of the receptor. Complement activation on the muscle membrane surface also decreases expression of AChRs on the muscle cell. This results in weakness of skeletal muscles, and almost always includes eye muscles (79).

#### The Role of Complement in MG

Immunohistologic examination of the post-synaptic membranes from patients with MG demonstrates deposition of immunoglobulin and C3 in nearly all cases (80). C5b-9 has also been detected at this location (81). C5 deficiency was protective in a passive model of the disease in rats. In a mouse model of MG, C4 and C5 deficiency were both similarly protective, indicating that disease is caused through activation of the classical pathway by pathologic antibodies and is mediated by C5a or C5b-9 (82, 83). Conversely, deficiency of CD55 or CD59 exacerbates disease, indicating a role for these complement regulators in attenuating injury (84).

Several therapeutic complement inhibitors have shown efficacy in pre-clinical models. A soluble CR1 construct was protective in the rat models (85), and complement blockade with monoclonal antibodies to C5 or C6 has also proven effective in rodent models (86, 87).

#### Therapeutic Complement Inhibitors in MG

Based on the promising pre-clinical data, eculizumab has studied in several clinical trials of patients with relapsing or refractory MG. A phase 2 placebo controlled study showed that treatment with the drug was associated with improved muscle strength (88). This was followed by a randomized, double-blind, phase 3 study (REGAIN) (89). In this study, 126 patients were randomized to eculizumab or placebo for 26 weeks. The primary endpoint for the study was the Myasthenia Gravis-Activities of Daily Living (MG-ADL) score. This endpoint was not significantly different in the eculizumab group when evaluated by worst-rank ANCOVA. There was a significant improvement when pre-specified secondary endpoints were evaluated, however. Based on these clinical studies, eculizumab has been approved in the United States, Europe, and in Japan for treatment of MG (90). As more MG patients are treated with complement inhibitors the benefits and limitations of this approach will become clearer.

### Neuromyelitis Optica

Neuromyelitis optica (NMO) is an autoimmune disease of the nervous system caused by autoantibodies, usually to aquaporin 4 expressed on astrocytes (91). Antibody deposition on the cells leads to demyelination. NMO primarily affects the optic nerve and spinal cord, but it can also affect the cerebral cortex. Immunosuppressive drugs are beneficial in some cases, and therapeutic plasma exchange ameliorates disease by removing the pathogenic autoantibodies.

#### The Role of Complement in NMO

Complement proteins, including C1q, C3, C4, and C5b-9, are deposited in nearly all cases of active NMO (92, 93). In animal models, the injection of AQP4-IgG alone is insufficient to initiate demyelination, but intracerebral co-injection of the antibodies with human complement sufficient serum induces lesions with the seminal histopathologic features of NMO (94). In the absence of complement or in the presence of complement inhibition, on the other hand, there is no evidence astrocyte or oligodendrocyte loss. In a rat model, peripherally administered AQP4-IgG caused NMO-like lesions in rats that received an intracerebral needle injury, to permit passage of the antibodies into the central nervous system (95). Injury was prevented, however, by depletion of complement with cobra venom factor (95). Similarly, in mice, it has been shown that lesions induced by injection of anti AQP4 autoantibodies require complement activation by the immunoglobulin (96).

#### Therapeutic Complement Inhibitors in NMO

Eculizumab successfully reduced the number of disease flares in an open label study that included 14 patients with NMO who had previously had relapsing disease (97). Patients were treated with the drug for 1 year. During that period 12 of the patients did not have any disease flares, and the other two patients had only single, minor flares. A phase 3 randomized controlled double-blind trial to test the efficacy of eculizumab in patients with relapsing disease (PREVENT study) recently completed its recruitment phase. This study enrolled 143 seropositive patients, who had had active disease within the previous 2 years.

## Conclusions

The complement system is activated in almost all antibody-mediated autoimmune diseases. Therefore, drugs that block complement activation may block downstream mediators of injury that are common to most, if not all, of these diseases. The complement cascade plays a central role in the immune response. Complement inhibition may rapidly reduce tissue inflammation and damage, and it may also attenuate T cell and B cell activation, thereby reducing autoimmunity (11). Most ongoing trials are using complement inhibitors to block acute tissue inflammation. As these studies are analyzed, however, it will be interesting to see whether an effect on autoantibody titers can also be detected.

The complement system is activated by antibodies in the autoimmune diseases discussed above, and activation usually occurs through the classical pathway. In some of these diseases, such as MN, activation may occur through the alternative or lectin pathways. Furthermore, the classical pathway may help to eliminate nuclear antigens in diseases such as lupus. Thus, there is a rationale to develop and test inhibitors that can specifically block these other pathways or specific downstream complement activation fragments, such as C5a. Many new complement inhibitory drugs are currently in development (12, 13). As these drugs enter the clinic, it will be fascinating to test whether the benefits of complement inhibition are similar among the various autoimmune diseases, or whether disease specific approaches will be needed.

## Author Contributions

JT contributed to the conception, writing, and editing of this manuscript. RY contributed to the conception and writing of this manuscript.

### Conflict of Interest Statement

JT receives royalties from Alexion Pharmaceuticals, Inc. JT is also a consultant for AdMIRx, Inc., a company developing complement inhibitors. He holds stocks and will receive royalty income from AdMIRx. The remaining author declares that the research was conducted in the absence of any commercial or financial relationships that could be construed as a potential conflict of interest.
